# Targeted transcriptomic analysis of well-differentiated and dedifferentiated liposarcoma reveals multiple dysregulated pathways including glucose metabolism, TGF-β, and HIF-1 signaling

**DOI:** 10.3389/fonc.2024.1456071

**Published:** 2024-11-26

**Authors:** Ashley Patton, Natalie Horn, Puja Upadhaya, Patricia Sarchet, Raphael E. Pollock, Steve Oghumu, Obiajulu Hans Iwenofu

**Affiliations:** ^1^ Department of Pathology and Laboratory Medicine, The Ohio State University Medical Center, Columbus, OH, United States; ^2^ Comprehensive Cancer Center, The Ohio State University Medical Center, Columbus, OH, United States; ^3^ Department of Surgery, Division of Surgical Oncology, The Ohio State University Medical Center, Columbus, OH, United States

**Keywords:** dedifferentiated liposarcoma, well-differentiated liposarcoma, NanoString, TGF-β, transcriptome, glucose metabolism, HIF, signaling pathways

## Abstract

Liposarcoma is the most prevalent sarcoma in adults representing 20% of all sarcomas with well-differentiated/dedifferentiated among the most common subtypes represented. Despite multimodality treatment approaches, there has not been any appreciable change in survival benefit in the past 10 years. The future of targeted therapy for WD/DDLPS is promising with the intention to spare multi-visceral removal due to radical surgical resection. Therefore, there is a need to expand upon the molecular landscape of WDLPS and DDLPS which can help identify potential therapeutic targets for the treatment of this disease. Targeted transcriptome analysis using the NanoString tumor signaling 360 panel revealed a dysregulation in glucose metabolism and HIF1 signaling pathways in both WDLPS and DDLPS when compared to normal fat controls. WDLPS, however, demonstrated upregulation of *HIF-1A* and *TGF-β* when compared to DDLPS by targeted transcriptome analysis and orthogonal validation by RT-qPCR suggesting activation of EMT pathway in WDLPS when compared to DDLPS. Our findings implicate a putative role for dysregulation in glucose metabolism, TGF-β and HIF1 signaling in the pathogenesis of both WD/DDLPS suggesting a possible proinflammatory tumor environment within WDLPS and subsequent activation of the TGF-β signaling pathway.

## Introduction

Liposarcoma is one of the prevalent mesenchymal neoplasms in adults, representing 20% of all sarcomas and is divided into 5 main subtypes including atypical lipomatous tumor (ALT)/well-differentiated liposarcoma (WDLPS), dedifferentiated liposarcoma (DDLPS), myxoid liposarcoma, pleomorphic liposarcoma, and atypical spindle cell/pleomorphic lipomatous tumor. Unique molecular and biological differences exist that define the histopathologic/clinical subtypes. The largest subgroup includes both ALT/WDLPS and DDLPS representing approximately 45% of all liposarcomas (1–3). When localized to the extremities, the term atypical lipomatous tumor is used, whilst liposarcoma is used for retroperitoneal and central body cavity locations.

The morphology and clinical behavior of WDLPS and DDLPS differ drastically, and the biological behavior of DDLPS portends a worse prognosis due to its aggressive nature, high local recurrence rate, and ability to metastasize (4–7). We also recognize that 90% of DDLPS arises *de novo* in the absence of a WDLPS precursor lesion. (8) The non-adipogenic morphology of DDLPS is distinct from WDLPS, often consisting of a high-grade non-lipogenic spindle cell sarcoma (7). In cases of secondary DDLPS the dedifferentiation of WDLPS is not well understood. Moreover, both WDLPS and DDLPS are among the most common retroperitoneal sarcoma establishing a critical need to further elucidate the molecular landscape that defines tumorigenesis in retroperitoneal liposarcoma and to understand the underlying mechanisms that distinguish the unique biological behavior of WDLPS and DDLPS. For the purposes of uniformity, we will restrict our terminology to WDLPS rather than ALT.

Previous studies on molecular profiling of lipomatous tumors have established amplification of *MDM2* as a diagnostic marker distinguishing WDLPS and DDLPS from benign lipomatous tumors as well as other subtypes of liposarcoma (9, 10). Both WDLPS and DDLPS harbor supernumerary ring and giant marker chromosomes resulting from amplification of the 12q13-15 region containing the *MDM2* locus. The MDM2-p53 axis has been well established in summarizing the regulatory mechanisms by which MDM2 functions to facilitate p53 degradation by E3 ubiquitin ligase thereby inhibiting the tumor suppressor activity of p53 (11, 12). Further, overexpression of neighboring genes, *CDK4* and *HMGAI*, have been identified in WDLPS and DDLPS as well as exploited as potential molecular targets for treatment (10). However, the molecular genetic determinants of transformation of WDLPS to DDLPS are not known.

The landscape of systemic and molecular-based therapies for WDLPS and DDLPS are limited to CDK4 and PDL1 inhibitors. Small molecule MDM2 (i.e. nutlins, Siremadlin) and cdk4 inhibitors (ribociclib) have shown limited efficacy in phase I clinical trials (13, 14). Novel targets of the EMT pathway are emerging as potential therapeutics for the treatment of DDLPS. For example, CCNDBP1, a tumor suppressor, has been shown to suppress gene expression and protein levels of key regulators of the EMT *in vivo*. (15).

Surgical resection is currently the standard treatment. (16) Chemotherapy used in the adjuvant setting for DDLPS has shown no significant improvement in clinical outcomes regarding local recurrence. More so, the overall 5-year survival rate of DDLPS is approximately 40-60% and no significant improvement in survival rates has been achieved in the past 15 years. (17) Despite surgical resection, retroperitoneal WDLPS recurs in approximately 20% of cases and has a higher incidence of dedifferentiation to DDLPS with an increased 20-year mortality rate between 30-40% (2, 18). Cytotoxic chemotherapy is not routinely utilized in patients with retroperitoneal WDLPS indicating a critical need to identify therapeutic targets for the treatment of this aggressive subtype of WDLPS. Importantly, the cellular pathways that define the sarcomagenesis of WDLPS and DDLPS and distinguish each entity continue to remain elusive. Thus, there remains an urgent need for more targeted therapeutic options for WD/DDLPS patients.

Using the targeted NanoString 360 tumor signaling transcriptomic panel, we further explore the molecular underpinnings of WDLPS and DDLPS and identify key cellular pathways unique to the two liposarcoma subtypes. Expanding our understanding of the molecular drivers in WDLPS may lead to the identification of therapeutic targets for its treatment to prevent recurrence.

## Materials and methods

### Sample selection

Snap-frozen tissue samples were retrieved from tissue biorepository, obtained at the time of radical surgical resections. All the patients were chemoradiation therapy naïve, and the nature of the surgical resections were R1.

Selection criteria included a known diagnosis of retroperitoneal WDLPS [n=12 (males = 7; females = 5); median age = 61.5 years] and DDLPS [n=12 (males = 8; females = 4); median age = 61.5 years]. Normal fat was collected as normal controls [n = 4 (males =3; females = 1); median age = 59 years] (**Supplementary Table 1**).

### Quantitative real-time PCR analysis

Total RNA was isolated from snap-frozen human tissue samples using RNeasy Lipid Tissue Mini Kit according to manufacturer’s protocol (Qiagen). Preparation of cDNA was achieved using the high-capacity cDNA reverse transcription kit with RNase Inhibitor (Applied Biosystems, Thermo Fischer Scientific). SYBR Green (Bio-Rad) biochemistries was used to perform qRT-PCR to quantify gene expression with *GAPDH* or *ACTB* as reference genes.

### NanoString nCounter gene expression assay

NanoString nCounter Tumor Signaling 360 panel was used for direct detection of target molecules using color-coded molecular barcodes, providing a digital simultaneous quantification of the number of target molecules. The panel included standard probes for the Tumor Signaling 360 panel without customization. A concentration of 100 ng of total RNA was hybridized overnight with nCounter Reporter (8 μL) probes in hybridization buffer and in excess of nCounter Capture probes (2 μL) at 65°C for 18 h. After overnight hybridization, probe excess was removed using two-step magnetic bead-based purification on an automated fluidic handling system (nCounter Prep Station). Biotinylated capture probe-bound samples were immobilized and recovered on a streptavidin-coated cartridge. The abundance of specific target molecules was then quantified using the nCounter digital analyzer. Individual fluorescent barcodes and target molecules present in each sample were recorded with a CCD camera by performing a high-density scan (325 fields of view). Images were processed internally into a digital format and exported as Reporter Code Count (RCC).

### Western Blot

A total of 20 µg of protein was loaded into a 10% Tris-HCL gel. Proteins were transferred to a PVDF membrane (0.2 µm). The PVDF membrane was blocked with 5% nonfat dry milk in 1X TBS-T (1% polysorbate 20) for 1 hour and incubated with the primary antibody, rabbit anti-human TGF-β (Cell Signaling, Danvers, MA, USA, #3711) or rabbit anti-human GAPDH (Cell Signaling, 2118S) overnight at 4°C. Membranes were incubated in a secondary goat anti-rabbit HRP-linked antibody (Thermo Fisher Scientific, 31460, Rockford, IL, USA) for 2 hours. Membranes were incubated with chemiluminescence ECL Western Blotting substrate (ThermoScientific, Waltham, MA, USA). ImageJ was used to quantify the protein intensity of TGF-β relative to GAPDH.

### Statistical analysis

Statistical analysis was performed using GraphPad Prism 9.5.1 (GraphPad Software, San Diego, CA, USA). Statistical differences were determined using a one-way ANOVA followed by a Tukey-Kramer or Bonferroni test for *post hoc* comparison. Nanostring gene expression was performed by using ROSILAND^®^ software. In brief, ROSILAND^®^ software follows the nCounter^®^ Advanced Analysis protocol for normalization. Fold changes and p-values were calculated using the nCounter^®^ Advanced Analysis 2.0. The adjusted p-value was calculated using the Benjamini-Hochberg method of estimating the false discovery rate (FDR).

## Results

### WDLPS and DDLPS demonstrate a global differential gene expression and downregulation in genes associated with glucose metabolism compared to normal fat

**Figure 1:**
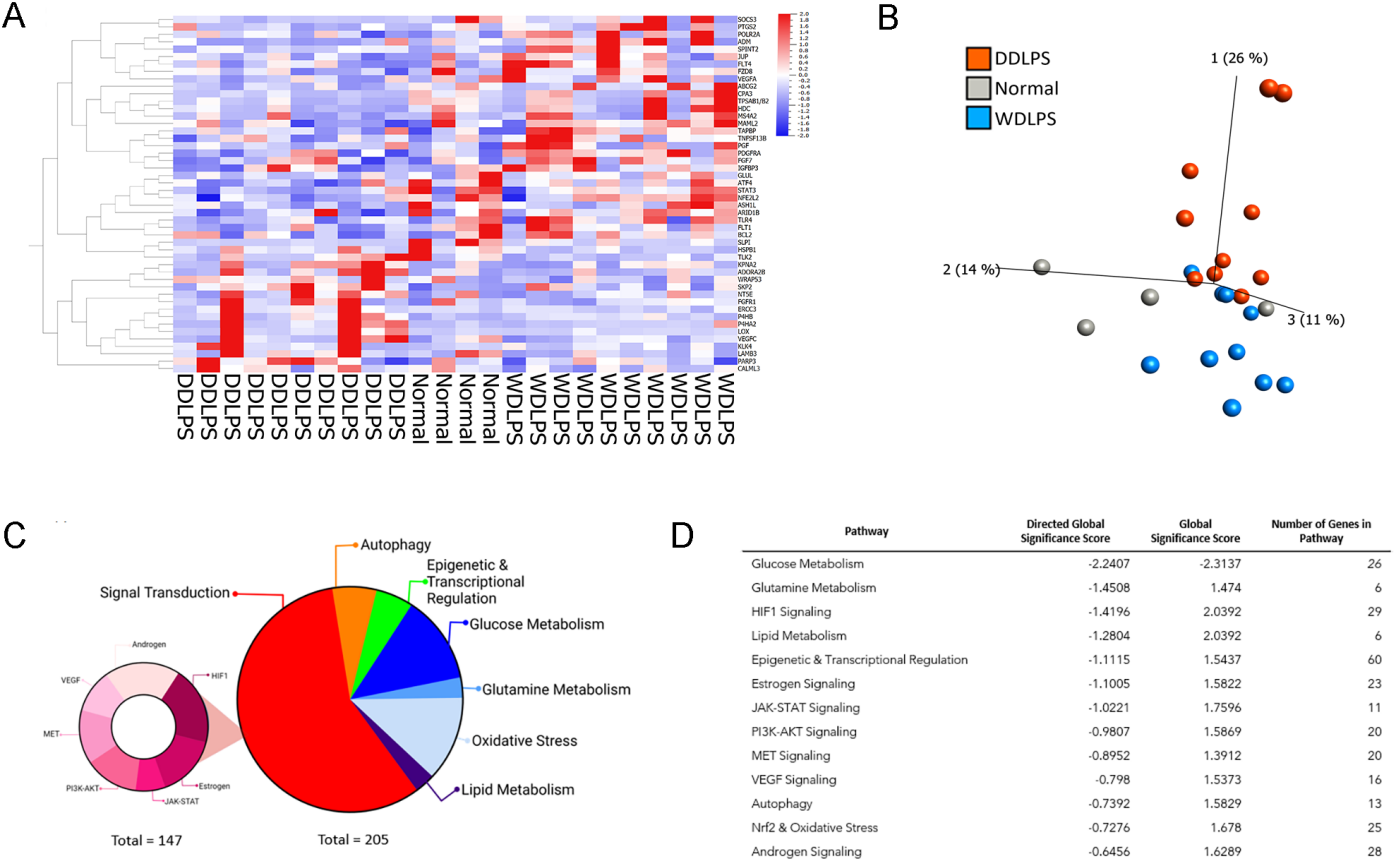
NanoString nCounter Tumor Signaling 360 Panel was used to characterize the gene expression profiles and pathway analysis of WDLPS, DDLPS, and the normal fat control. **(A, B)** Heat map **(A)** Principal component analysis **(B)** generated by hierarchical clustering analysis to represent the unique expression profiles for WDLPS, DDLPS, and normal fat. Global pathway analysis and differential gene expression show differentially expressed genes involved in a variety of biochemical pathways including signal transduction, metabolism, autophagy, and epigenetic and transcriptional regulation (Global significance cutoff scores of 1.0 and -1.0) **(C, D)**.

The NanoString nCounter Tumor Signaling 360 Panel was used to characterize the gene expression profiles of WDLPS, DDLPS, and the normal fat control. The heat-map (**Figure 1A**) and principal component analysis (**Figure 1B**) show global differential gene expression and clustering between well differentiated liposarcoma, dedifferentiated liposarcoma and normal fat. The panel analyzed 760 genes involved in global pathways including tumorigenesis, invasion, metastasis, angiogenesis, immune evasion, inflammation and glucose metabolism (**Figures 1C, D**). Our initial evaluation revealed 352 differentially expressed genes involved in a variety of biochemical pathways including signal transduction, metabolism, autophagy, and epigenetic and transcriptional regulation (**Figure 1C**). Of the 352 differentially expressed genes between both the liposarcoma groups (WDLPS and DDLPS) and normal fat, 147 genes were involved in signal transduction pathways that function to regulate cell cycle progression, immune response, autophagy, and oxidative stress (**Figures 1C, D**). Glucose metabolism demonstrated a global downregulation in both WDLPS and DDLPS when compared to normal fat (global significance score >2.0) as highlighted in **Figure 1D**. Additionally, glutamine metabolism and HIF-1 signaling pathways were upregulated in both WDLPS and DDLPS thereby suggesting a metabolic shift in WDLPS and DDLPS when compared to the nonneoplastic normal fat control (**Figure 1D**).

**Figure 2:**
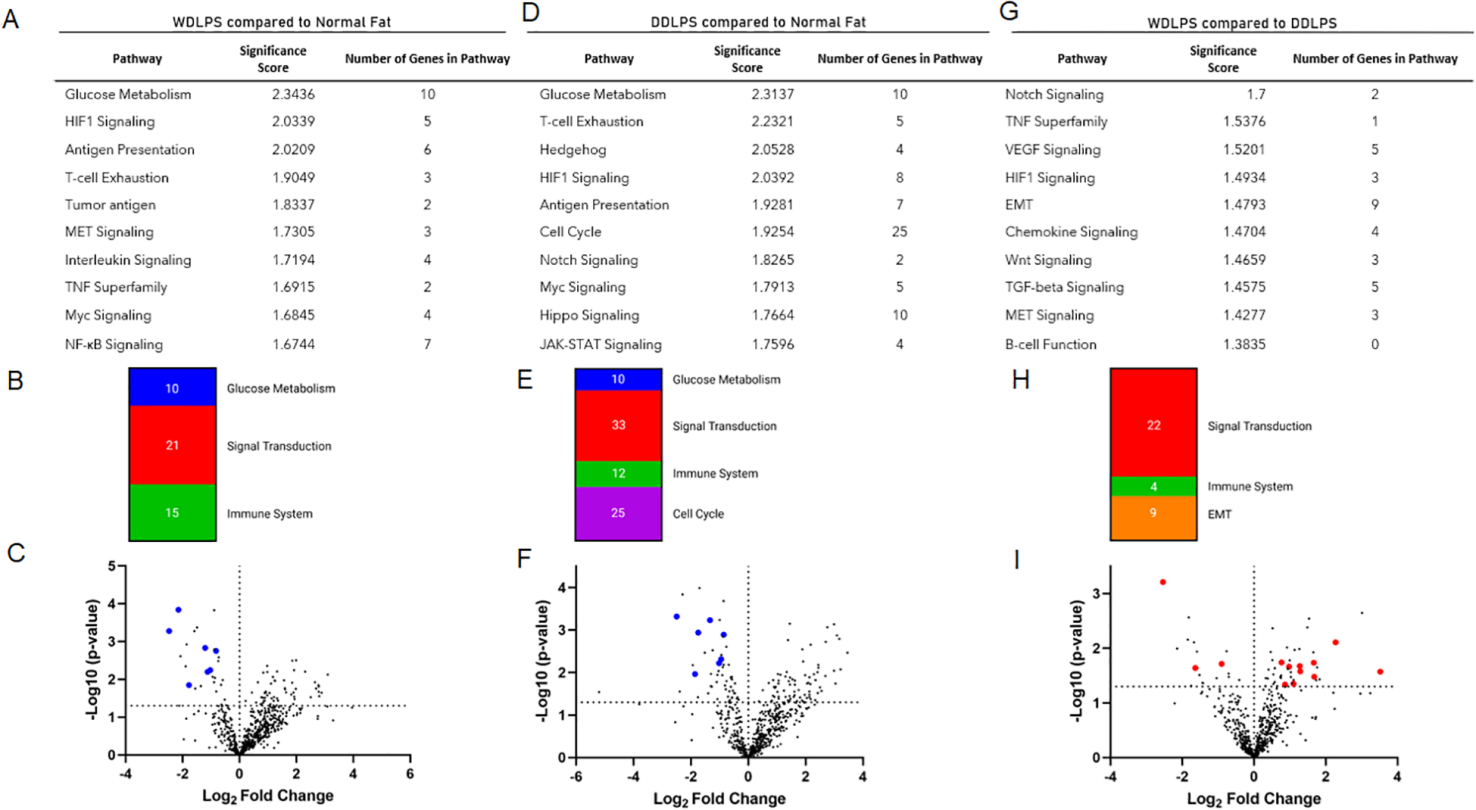
NanoString nCounter Tumor Signaling 360 Panel was used to characterize the gene expression profiles and pathway analysis of WDLPS, DDLPS, and the normal fat control. **(A, B)** Significantly altered pathways in WDLPS compared to normal fat (significance score cutoff 1.0). **(C)** Volcano plot demonstrating the distribution of differential gene expression for WDLPS and normal fat. **(D, E)** Significantly altered pathways in DDLPS compared to normal fat (significance score cutoff 1.0). **(F)** Volcano plot demonstrating the distribution of differential gene expression for DDLPS and normal fat. **(G, H)** Significantly altered pathways in WDLPS compared to DDLPS (significance score cutoff 1.0). **(I)** Volcano plot demonstrating the distribution of differential gene expression for WDLPS and DDLPS; blue and red dots correspond to genes associated with glucose metabolism and signal transduction, respectively.

Targeted transcriptomic analysis of WDLPS and normal fat tissue identified 82 differentially expressed genes representing significantly altered cellular pathways. Among the pathways with the greatest significance score include glucose metabolism and HIF-1-signaling (downregulated) as well as antigen presentation (upregulated) with significance score >2.0 (**Figures 2A–C**). Our initial comparison between DDLPS and normal fat in **Figures 2D–F** revealed 118 differentially expressed genes representing significantly altered cellular pathways (significance score >2.0) including glucose metabolism (downregulated) as well as HIF-1signaling, T-cell exhaustion, hedgehog (upregulated). The comparison between WDLPS and DDLPS (**Figures 2G–I**) shows differential gene expressions involved in the Notch, TNF and VEGF-signaling pathways, though the significance scores are <2.0.

**Figure 3:**
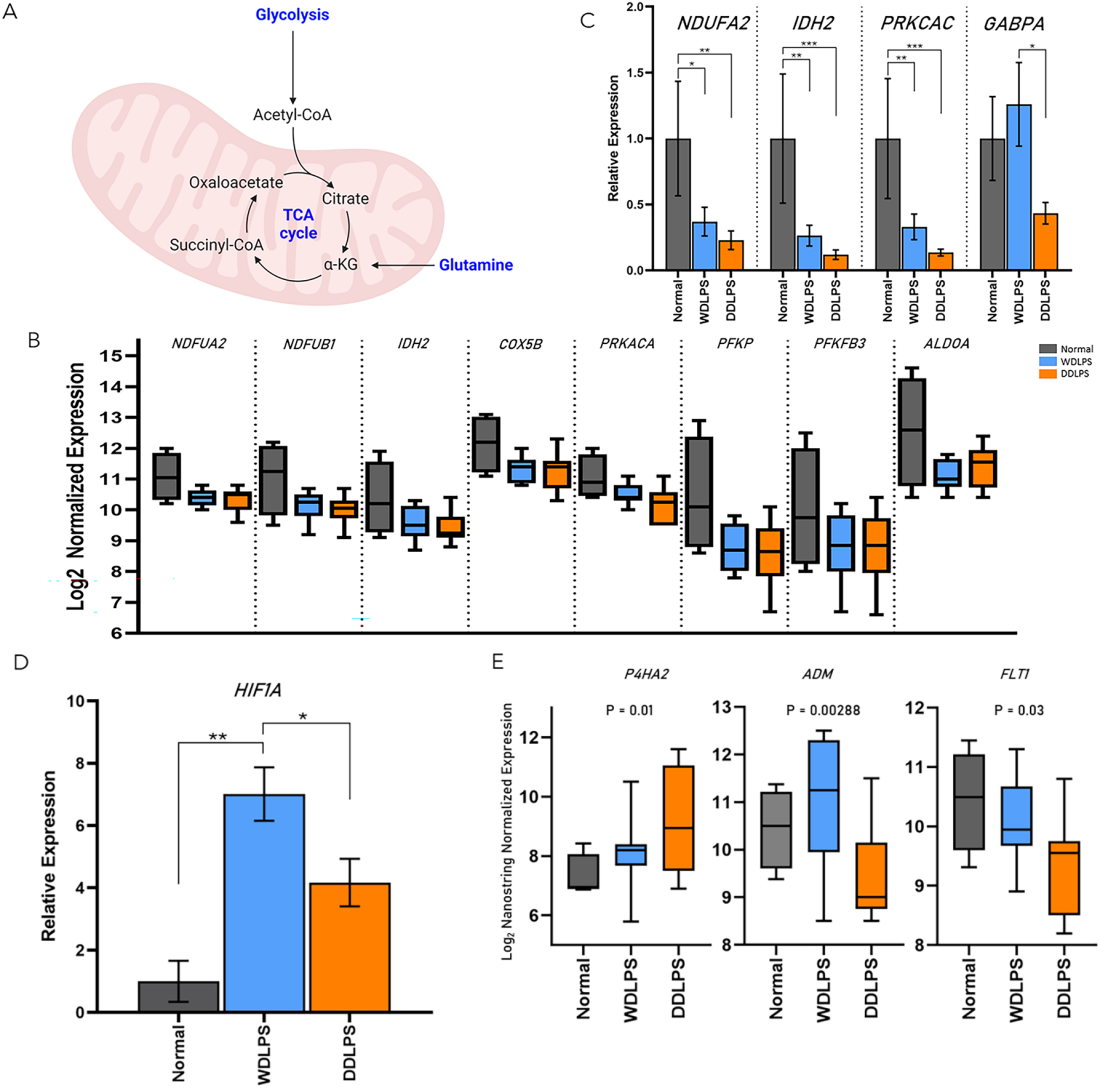
WDLPS and DDLPS demonstrate a global downregulation in genes associated with glucose metabolism and upregulated HIF1 signaling. **(A)** Overview of glycolysis. **(B)** Differentially expressed genes involved in glucose metabolism (padj < 0.05; log2 fold change > 1 & < -1). **(C)** Real-time PCR demonstrating a downregulation of *NDUFA2, IDH2, PRKCAC* in both WDLPS and DDLPS when compared to normal fat as well as downregulation of *GABPA* in DDLPS when compared to WDLPS and normal fat. **(D)** HIF1A expression is upregulated in WDLPS when compared to DDLPS and normal fat. **(E)** Differential expression of genes associated with HIF1-signaling pathway (padj < 0.05; log2 fold change > 1 & < -1). * represents p<0.05; ** represents p<0.01; and *** represents p<0.001.

Significantly downregulated genes in both WDLPS and DDLPS when compared to normal fat were *PFKP, ALDOA, PKM, PFKFB3, LDHA, NDUFB1, IDH2, COX5B, NDUFA2, and PRKACA* which share key modulators of glucose metabolism, specifically aerobic respiration including glycolysis and the electron transport chain (**Table 1**; **Figures 3A–C**).

**Table 1:** Differentially expressed genes involved in glucose metabolism.

Gene	Full Name	Cellular Pathway	Key Function of Encoded Protein	Fold Change	P-value
*NDUFA2*	NADH: ubiquinone oxidoreductase subunit A2	Glucose Metabolism	Subunit of the NADH: ubiquinone oxidoreductase (complex I), the first enzyme complex in the electron transport chain within the inner mitochondrial membrane	-1.81066	0.00128
*NDUFB1*	NADH: ubiquinone oxidoreductase subunit B1	Glucose Metabolism	Involved in mitochondrial respiratory chain complex I assembly	-2.52667	0.00059
*IDH2*	Isocitrate dehydrogenase (NADP(+))2	Glucose Metabolism	NADP (+) –dependent isocitrate dehydrogenase within the mitochondria that catalyzes the oxidative decarboxylation of isocitrate to 2-oxoglutarate	-2.36756	0.003
*COX5B*	Cytochrome c oxidase subunit 5B	Glucose Metabolism	Component of the cytochrome c oxidase, the last enzyme in the mitochondrial electron transport chain which drives oxidative phosphorylation	-0.02546	0.00607
*PRKACA*	Protein kinase cAMP-activated catalytic subunit alpha	Glucose Metabolism	One of the catalytic subunits of protein kinase A and is involved in a variety of cellular processes including glucose metabolism, cell division, and apoptosis	-1.92719	0.00486
*PFKP*	Phosphofructokinase, platelet	Glucose Metabolism	Platelet type isoform of PFK that initiates the first committing step of glycolysis by catalyzing the phosphorylation of D-fructose 6-phosphate to fructose 1,6-bisphosphate by ATP	-5.64334	0.00048
*PFKB3*	6-phosphofructo-2kinase/fructose-2,6-bisphosphatase 3	Glucose Metabolism/HiF-1 Signaling	A bifunctional protein involved in the synthesis and degradation of fructose-2,6-bisphosphate to regulate glycolysis, cell cycle progression, and apoptosis	-3.62025	0.01092
*ALDOA*	Aldolase dehydrogenase 1A (class 1), alpha polypeptide	Glucose Metabolism/HiF-1 Signaling	Catalyzes the reversible conversion of fructose-1,6-bisphosphate to glyceraldehyde 3-phosphate and dihydroxyacetone phosphate	-3.35627	0.00115

Both WDLPS and DDLPS displayed reduced aerobic glucose metabolism when compared to normal fat. Downregulated genes involved in glucose metabolism identified in the DDLPS group were similar to genes identified in WDLPS which included *PFKP, PFKFB3, IDH2, PRKACA, ALDOA, COX5B, NDUFB1*, and *NDUFA2* (**Figures 3A–C**). Real-time PCR was performed to validate differential gene expression in WDLPS, DDLPS, and normal fat. **Figures 3B, C** highlights decreased gene expression of *NDUFA2*, *IDH2*, and *PRKCAC* in both WDLPS and DDLPS when compared to normal fat tissue. Among the genes involved in glucose metabolism include *PFKP, PFKFB3, IDH2*, and *PRKACA* which are known to serve multifactorial roles in carcinogenesis including differentiation, cell-cycle regulation, and apoptosis however, the role of these genes in sarcomagenesis of WDLPS and DDLPS is not well elucidated.

### Glutamine synthesis is altered in DDLPS when compared to WDLPS and normal fat

One means by which cancer metabolism is altered is by utilizing a variety of different biosynthetic substrates including glutamine. Glutamine synthesis was reduced in both WDLPS and DDLPS when compared to normal fat potentially supporting a metabolic shift toward glutaminolysis. Interestingly, GA binding protein transcription factor subunit alpha, *GABPA*, was downregulated in DDLPS when compared to normal fat and WDLPS and confirmed by RT-PCR (**Figure 3C**). However, the role of *GABPA* in the pathogenesis of DDLPS is still unknown.

### The HIF-1 signaling pathway is upregulated in WDLPS

The HIF-1 pathway was significantly altered in both WDLPS and DDLPS when compared to the normal fat control (**Figures 3D, E**). Expression of *HIF-1A* was significantly upregulated in WDLPS and DDLPS compared to normal fat (**Figure 3D**) and confirmed by RT-PCR. Further exploration of the HIF-1A pathway revealed differences in the transcriptome profile of WDLPS and DDLPS (**Figure 3E**). Expression of *ADM* and *FLT1*, genes also involved in angiogenesis, were increased in WDLPS when compared to DDLPS (**Figure 3E**). In contrast, DDLPS showed an upregulation of *P4HA2*, a key mediator of collagen synthesis when compared to WDLPS (**Figure 3E**).

### Profiling of resident immune cells reveals an increased population of T-cells in WDLPS

**Figure 4:**
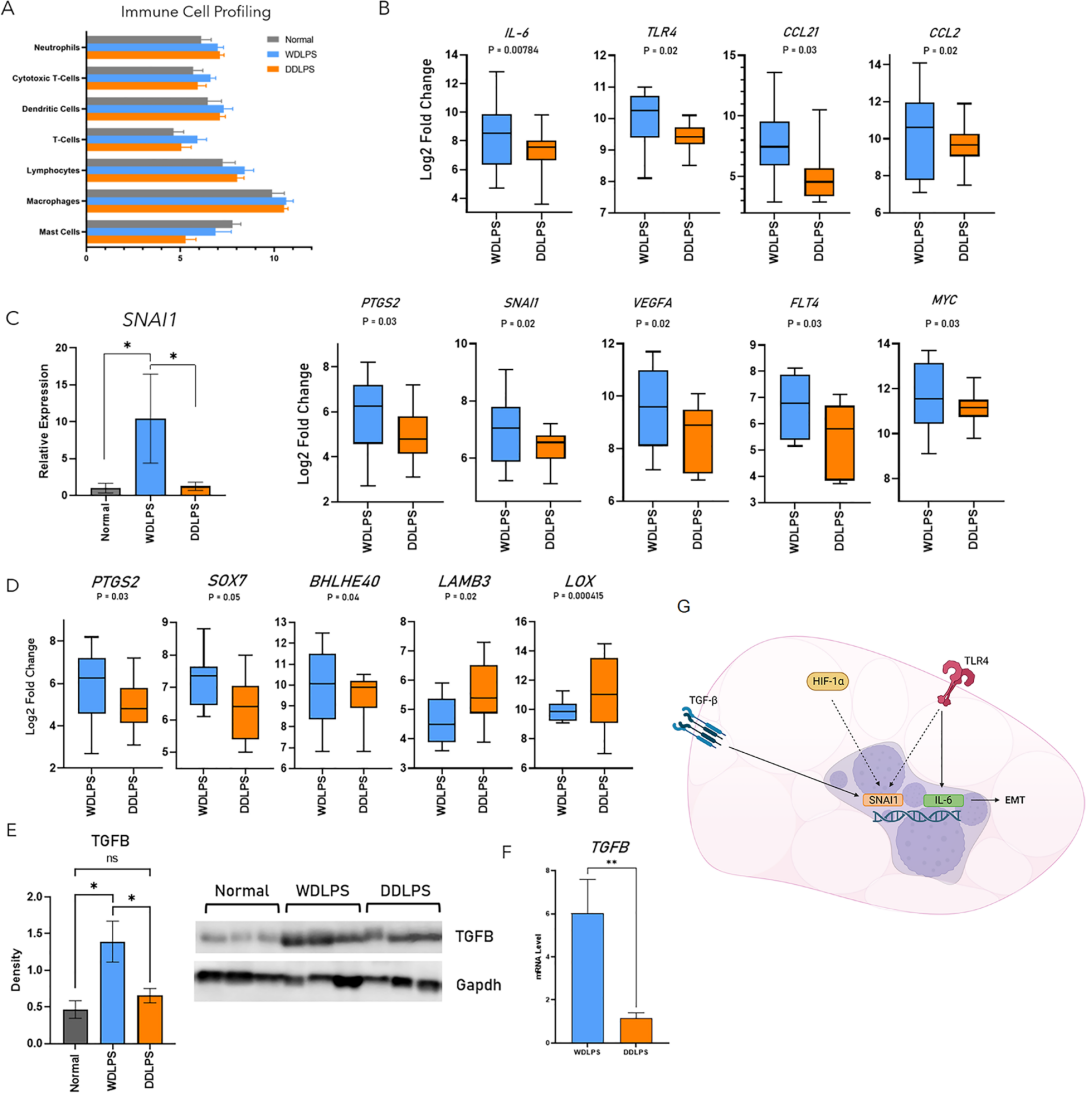
Profiling of resident immune cells reveals an increased population T-cells and upregulation of the EMT pathway in WDLPS. **(A)** Cell profiling of resident immune populations based on NanoString nCounter Tumor Signaling 360 Panel capture probes demonstrates increased lymphocytes, specifically T-cell and cytotoxic T-cells in WDLPS compared to DDLPS and normal fat. Neutrophils are increased in WDLPS and DDLPS compared to normal fat. **(B)** Differential expression of genes involved in the EMT pathway demonstrates upregulation of *IL-6, TLR4, CCL21, CCL2, PTGS2, SNAI1, VEGFA, FLT4*, and *MYC* when compared to DDLPS (padj < 0.05; log2 fold change > 1 & < -1). **(C)** SNAI1 is upregulated in WDLPS when compared to DDLPS and normal fat by real-time PCR. **(D)** Genes (*PTGS2, SOX7, BHLHE40, LAMB3, LOX)* downstream of TGF-β signaling are upregulated in WDLPS when compared to DDLPS. **(E, F)** TGF-β protein and mRNA levels **(E)** are increased in WDLPS confirmed by Western blot and real-time qPCR (*p-value < 0.05). **(G)** Summary of EMT-related pathways activated in WDLPS. ** represents p<0.01.

Resident immune cells serve a predominantly anti-inflammatory role within normal adipose tissue as well as indirectly regulating adipocyte metabolism. Therefore, as an additional metric, we evaluated the composition of the immune fraction within the tumor microenvironments of WDLPS and DDLPS as well as normal adipose tissue using Nanostring cell profiling capabilities (**Figure 4**). The population of neutrophils was elevated in both WDLPS and DDLPS when compared to the normal fat control (**Figure 4A**). Immune profiling revealed increased lymphocytes, specifically T-cells and cytotoxic T-cells, in the WDLPS samples when compared to both DDLPS and normal adipose tissue (**Figure 4A**). Populations of the remaining immune cells including dendritic cells, macrophages, and mast cells remained unchanged (**Figure 4A**).

### Epithelial-mesenchymal transition and TGF-β pathways are upregulated in WDLPS

Our analysis established that WDLPS and DDLPS share similar metabolic profiles, specifically a shift from glucose metabolism to glutaminolysis thus exhibiting a cancer-promoting metabolic phenotype. Of the 760 differentially expressed genes, 47 genes were significantly altered in WDLPS when compared to DDLPS (**Figure 1**). Interestingly, the corresponding cellular pathways significantly altered in WDLPS when compared to DDLPS are predominantly associated with metastasis including inflammation, angiogenesis, and extracellular matrix remodeling (**Figures 4B–D**). Our data revealed that genes involved in metastasis, specifically pathways that comprise the epithelial-mesenchymal transition, EMT, pathway were differentially expressed in the WDLPS and DDLPS (**Figures 4B–D**). It is well known that the biological behavior of DDLPS is more aggressive than WDLPS and can metastasize to distant sites. The behavior of WDLPS, however, is more insidious as it tends to expand in the peritoneal and retroperitoneal cavities engulfing visceral organs. Genes involved in the EMT pathway including proinflammatory cytokines/chemokines *IL-6, CCL21, CCL2*, as well as the innate immune receptor, *TLR4*, were upregulated in WDLPS when compared to DDLPS (**Figure 4B**). Additionally, genes involved in angiogenesis and vascular permeability including *VEGFA, FLT4, FLT1*, and *PTGS2* were upregulated in WDLPS when compared to DDLPS with the exception of *VEGFC* which showed increased mRNA levels in DDLPS when compared to WDLPS (**Figure 4B**). Snail family transcriptional repressor 1, *SNAI1*, is known to be involved in promoting the EMT pathway in human cancer and is a direct downstream target of the HIF-1 pathway as well as the TGF-β signaling pathway. Evaluation of differentially expressed genes involved in the EMT pathway revealed an upregulation of SNAI1 in the WDLPS when compared to DDLPS (**Figure 4B**). Measurement of *SNAI1* mRNA levels by real-time PCR confirmed that *SNAI1* is upregulated in WDLPS when compared to DDLPS as well as normal fat (**Figure 4C**).

The TGF-β signaling pathway plays an integral role in adipogenesis by promoting the differentiation from preadipocytes to mature adipocytes programmed to store lipids primarily in the form of triglyceride. The TGF-β signaling also plays a role in cell proliferation and apoptosis and has been implicated in a variety of human cancers serving as a therapeutic target. The gene expression profiles of both WDLPS and DDLPS demonstrated that genes involved in the TGF-β signaling pathway by direct or indirect downstream transcriptional regulation are predominately up-regulated in WDLPS when compared to DDLPS (**Figure 4D**). Gene expression of TGF-β has been shown to be upregulated in the DDLPS by *in vitro* and *in vivo* studies. It is conceivable that the upregulation of TGF-β may implicate proinflammatory cytokines and other activators of EMT that drive the tumorigenesis of WDLPS (**Figure 4G**). However, our data revealed that both TGF- β protein and mRNA levels were elevated in WDLPS when compared to DDLPS (**Figures 4E, F**) as well as several genes involved in the TGF-β pathway including *PTGS2*, and *SOX7* apart from *BHLHE40*, *LAMB3*, and *LOX* which were unchanged or down-regulated in WDLPS, respectively (**Figure 4D**).

## Discussion

The NanoSting nCounter panel has also been widely accepted as an efficient screening modality for various tumor types including soft tissue sarcomas with the goal of identifying key tumorigenic pathways to aid in the further understanding of tumor biology. (19–22) NanoString has demonstrated efficacy and cost-effectiveness for the identification and diagnosis of WDLPS and DDLPS in a predominantly retrospective series of low-grade adipogenic tumors when compared to MDM2 FISH. (23) More recently, the NanoString nCounter platform has been employed to evaluate the immune profile of DDLPS for targeted immunotherapy. (24) This the first pilot study to identify key metabolic signatures between WDLPS and DDLPS utilizing the NanoString nCounter Tumor Signaling 360 Panel.

WDLPS and DDLPS share the cytogenetic abnormality, specifically amplification of the 12q13-15 region that leads to subsequent diagnostic amplification of *MDM2* and *CDK4*. Previous cytogenetic studies have identified alternative amplification of 1p32 and 6q23 in DDLPS and not identified in WDLPS. (25) Located within the 1p32 and 6q23 loci, are the genes jun proto-oncogene (*JUN*) and mitogen-activated protein kinase kinase5 (*MAP3K5*). (26–28) Lineage-defining molecular mechanisms of WDLPS and DDLPS, however, remain unknown.

The NanoString nCounter Tumor Signaling 360 Panel was selected for targeted transcriptomic analysis due to the comprehensive coverage of genes involved in tumorigenesis, immune signaling, and microenvironment. Results of the current study have provided insight into additional molecular pathways that can be further explored to develop enhanced therapeutic modalities for retroperitoneal WDLPS and DDLPS including molecular pathways that are somewhat surprising in distinguishing WDLPS from DDLPS such as HIF-1 signaling, TGF-β signaling, and the epithelial-mesenchymal transition (EMT) pathways that are indicators of aggressive tumor behavior and metastasis.

We have established that both WDLPS and DDLPS have metabolic profiles generated from our targeted analysis that differ significantly from normal fat including both notably glucose and glutamine metabolism. Glucose is a primary focus in cancer metabolism, especially in the setting of decreased nutrient precursors in the tumor microenvironment. Cancer cells maintain high rates of glucose metabolism and oxidative phosphorylation to fulfill the high anabolic demand. (29, 30) One mechanism of particular interest is the Warburg effect in which a state of hypoxia or pseudohypoxia induced by the tumor microenvironment and rapid growth reduces oxidative phosphorylation while increasing glucose metabolism. (31, 32) The tricarboxylic acid (TCA) cycle remains intact to support tumor cell growth and is thought to be rewired to provide the building blocks for cancer metabolism. (33, 34)

Glutamine is involved in a variety of energy-generating biosynthetic pathways including cancer metabolism which has generated special interest to utilize glutamine metabolism as a potential therapeutic target. (35) Glutamine is the most abundant amino acid in circulation and provides a readily accessible carbon and nitrogen source for cellular metabolism. Cancer cells can exploit glutamine for anaplerotic pathways by inducing glutaminolysis which converts glutamine to the byproduct α-ketoglutarate for entry to the TCA cycle. (36) Targeting glutamine metabolism has been shown to slow growth in soft tissue sarcomas. (37) Inhibition of glutamine synthetase induced antiproliferative effects in sarcoma cell lines (rhabdomyosarcoma, fibrosarcoma, osteosarcoma) including the liposarcoma derived cell line (SW8_72_) In particular, liposarcoma cells were highly sensitive to the cytotoxic effects of antitumor enzyme L-asparaginase increasing glutamine synthetase levels and reducing cell proliferation. (38) However, the effects of glutamine metabolism are not well-elucidated in human liposarcoma. Results from the targeted transcriptome analysis revealed reduced glutamine synthesis in both the WDLPS and DDLPS samples when compared to the normal fat controls suggesting a tumor-induced metabolic shift from glutamine synthesis to glutaminolysis. Inhibitors of glutamine synthesis may be potential therapeutic options in the treatment of sarcomas that express high levels of glutaminase and are dependent on glutamine metabolism.

The HIF-1 signaling pathway is crucial for cancer cells to adapt to hypoxic stress, specifically intratumoral hypoxia and pseudohypoxia, and is considered a risk factor for poor prognosis in a variety of cancer types including retroperitoneal sarcomas (leiomyosarcoma, malignant peripheral nerve sheath tumor, WDLPS and DDLPS). HIF-1 is a heterodimer consisting of α and β subunits. (39) Downstream targets of HIF-1 include genes involved in angiogenesis (i.e. vascular endothelial growth factor, VEGF) (38)

Transforming growth factor β (TGF-β) has long been considered a multifactorial regulator of embryonic development and homeostasis by stimulating or inhibiting cell proliferation. Dysregulation of TGF-β has been implicated in metabolic disease promoting fibrosis and inflammation, and cancer. (40–42) In early tumorigenesis, TGF-β signaling is shifted toward tumor suppression. However, unregulated TGF-β expression in cancer cells overcomes the apoptotic/cell cycle control of TGF-β promoting tumor progression. The non-canonical pathway activates various pathways including PI3K, JNK, and ERK MAP kinases involved in transcriptional regulation. (43) The absence of key components of the TGF-β pathway suggest reduced invasive potential. (44, 45) Several genes involved in the TGF- β pathway were found to be elevated in DDLPS when compared to WDLPS suggesting a potential role in the progression of DDLPS. The therapeutic potential of TGF-β in liposarcoma was investigated to show that inhibition of TGF- β signaling reduced the growth and metastasis of liposarcoma cells in mice.

Activation of the TGF-β pathway can promote epithelial to mesenchymal transition (EMT) promoting a motile mesenchymal phenotype which is a characteristic of invasion and metastasis. (46, 47) Downstream targets of TGF-β such as *SNAI1* mediate the EMT pathway in a SMAD-dependent manner. (48) Nine genes involved in the EMT pathway were upregulated in WDLPS when compared to DDLPS including proinflammatory cytokines/chemokines (*IL-6, TLR4, CCL21, CCL2*, *PTGS2*), cell proliferation (*MYC*), and angiogenesis (*VEGFA, FLT1*, and *FLT4*). The expression of *SNAI1* was upregulated in WDLPS when compared to DDLPS. Although WDLPS demonstrates a predominantly adipose-like morphology in contrast to the high-grade spindle morphology of DDLPS, both WDLPS and DDLPS can show focal to extensive myxoid stroma, sclerosis, or inflammatory infiltrate. A study evaluating the degree of sclerosis in cases of retroperitoneal WDLPS showed that the degree of sclerosis and not myxoid or inflammation had the greatest impact on prognosis. Cases of minimally sclerotic WDLPS have a more favorable outcome when compared to cases with advanced sclerosis. (44) Although WDLPS is not considered to possess metastatic potential, these findings suggest a possible proinflammatory tumor environment within WDLPS and subsequent activation of the TGF-β signaling pathway (**Figure 4G**). Additional studies are warranted to understand the role of TGF-β in the pathogenesis of WDLPS.

In conclusion, our data suggests that WDLPS and DDLPS demonstrate diverse molecular and metabolic profiles. In an era of personalized medicine, some questions remain; what additional molecular aberrations account for the different behavior and prognosis of WDLPS and DDLPS, and how can these molecular pathways be targeted for therapeutic alternatives to surgical resection? Therapeutic agents have been developed and are currently in preclinical or clinical phases for pathways including glutamine metabolism and TGF-β signaling which have demonstrated efficacy, at least, *in vivo* in liposarcoma-derived cell lines. The future of targeted therapy for WDLPS is promising with the goal to spare multi-visceral removal as a consequence of radical surgical resection and prevention of dedifferentiation to DDLPS.

## Data Availability

The raw data supporting the conclusions of this article will be made available by the authors, without undue reservation.

## Supplementary material

Supplementary Table 1 Sample information and collection criteria.
