# Supplementary material for: Targeted transcriptomic analysis of well-differentiated and dedifferentiated liposarcoma reveals multiple dysregulated pathways including glucose metabolism, TGF-β, and HIF-1 signaling
Source: Front Oncol. 2024 Nov 26;14:1456071. doi: 10.3389/fonc.2024.1456071 (PMC11628955; doi:10.3389/fonc.2024.1456071)
Supplement: Supplementary Table 1 — Sample information and collection criteria. [file DataSheet1.pdf]

Patient Sample #	Gender	Age	Location	Diagnosis	MDM2 +
1	Male	60 years-old	Retroperitoneum	WDLPS	Y
2	Male	76 years-old	Retroperitoneum/Pelvis	WDLPS	Y
3	Male	60 years-old	Retroperitoneum	WDLPS	FISH not performed
4	Male	62 years-old	Retroperitoneum	WDLPS	Y
5	Female	42 years-old	Retroperitoneum	WDLPS	Y
6	Female	72 years-old	Retroperitoneum	WDLPS	Y
7	Male	69 years-old	Retroperitoneum	WDLPS	Y
8	Male	62 years-old	Retroperitoneum	WDLPS	Y
9	Female	67 years-old	Retroperitoneum	WDLPS	Y
10	Female	57 years-old	Retroperitoneum	WDLPS	Y
11	Male	56 years-old	Retroperitoneum	WDLPS	Y
12	Female	61 years-old	Retroperitoneum	WDLPS	Y
13	Male	76 years-old	Retroperitoneum	DDLPS	Y
14	Female	57 years-old	Retroperitoneum	DDLPS	Y
15	Male	55 years-old	Retroperitoneum	DDLPS	Y
16	Male	62 years-old	Retroperitoneum	DDLPS	Y
17	Female	64 years-old	Retroperitoneum	DDLPS	Y
18	Male	65 years-old	Groin	DDLPS	Y
19	Male	75 years-old	Retroperitoneum	DDLPS	Y
20	Female	60 years-old	Retroperitoneum	DDLPS	Y
21	Female	61 years-old	Retroperitoneum	DDLPS	Y
22	Male	64 years-old	Pelvis/periduodenum	DDLPS	Y
23	Male	57 years-old	Retroperitoneum	DDLPS	Y
24	Male	60 years-old	Colon	DDLPS	Y
25	Male	56 years-old	Unknown	Normal	N
26	Female	36 years-old	Unknown	Normal	N

27	Male	62 years-old	Unknown	Normal	N
28	Male	76 years-old	Unknown	Normal	N

Abbreviations: well-differentiated liposarcoma, WDLPS; dedifferentiated liposarcoma, DDLPS; yes, Y; no, N; fluorescent in situ hybridization, FISH
